# Fluphenazine dihydro­chloride dimethanol solvate

**DOI:** 10.1107/S1600536812008707

**Published:** 2012-03-10

**Authors:** Joanna Petrus, Rafał Petrus, Bogusława Czarnik-Matusewicz

**Affiliations:** a Faculty of Chemistry, University of Wroclaw, 14 F. Joliot-Curie St., 50-383 Wroclaw, Poland

## Abstract

In the title compound {systematic name: 1-(2-hy­droxy­eth­yl)-4-[3-(2-trifluoro­methyl-10*H*-phenothia­zin-10-yl)prop­yl]piperazine-1,4-diium dichloride dimethanol disolvate}, C_22_H_28_F_3_N_3_OS^2+^·2Cl^−^·2CH_3_OH, the dihedral angle between the planes of the two outer benzene rings of the tricyclic phenothia­zine system is 46.91 (13)°. The piperazine ring adopts a chair conformation. The crystal structure is stabilized by O—H⋯Cl, N—H⋯Cl, C—H⋯O, C—H⋯Cl and C—H⋯F hydrogen bonds and contacts.

## Related literature

For the properties of phenothia­zines, see: Ford *et al.* (1988 ▶); Ohlow & Moosmann (2011 ▶); Tsakovska & Pajeva (2006 ▶) and for the biological properties of fluphenazine, see: Gasiorowski *et al.* (2001 ▶); Szabó *et al.* (1999 ▶). For related structures, see: Dahl *et al.* (1986 ▶); Dutkiewicz *et al.* (2010 ▶); McDowell (1978 ▶, 1980 ▶); Yathirajan *et al.* (2007 ▶). For puckering parameters, see: Cremer & Pople (1975 ▶);. 
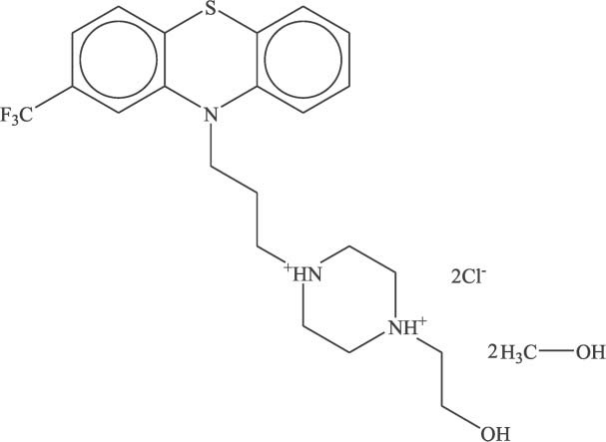


## Experimental

### 

#### Crystal data

C_22_H_28_F_3_N_3_OS^2+^·2Cl^−^·2(CH_4_O)
*M* *_r_* = 574.53
Orthorhombic, 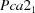

*a* = 39.76 (2) Å
*b* = 9.952 (8) Å
*c* = 7.127 (5) Å
*V* = 2820 (3) Å^3^
*Z* = 4
Mo *K*α radiation
μ = 0.35 mm^−1^
*T* = 85 K
0.24 × 0.02 × 0.01 mm

#### Data collection

Oxford Diffraction Xcalibur PX κ-geometry diffractometer with Onyx CCD camera
Absorption correction: multi-scan (*CrysAlis RED*; Oxford Diffraction, 2007 ▶) *T*_min_ = 0.850, *T*_max_ = 1.000
43952 measured reflections
13922 independent reflections
10615 reflections with *I* > 2σ(*I*)
*R*_int_ = 0.052

#### Refinement

*R*[*F* ^2^ > 2σ(*F* ^2^)] = 0.084
*wR*(*F* ^2^) = 0.197
*S* = 1.19
13922 reflections
330 parameters
1 restraint
H-atom parameters constrained
Δρ_max_ = 1.25 e Å^−3^
Δρ_min_ = −0.85 e Å^−3^
Absolute structure: Flack (1983 ▶), 5579 Friedel pairs
Flack parameter: 0.09 (7)

### 

Data collection: *CrysAlis CCD* (Oxford Diffraction, 2007 ▶); cell refinement: *CrysAlis CCD*; data reduction: *CrysAlis RED* (Oxford Diffraction, 2007 ▶); program(s) used to solve structure: *SHELXS97* (Sheldrick, 2008 ▶); program(s) used to refine structure: *SHELXL97* (Sheldrick, 2008 ▶); molecular graphics: *XP* in *SHELXTL* (Sheldrick, 2008 ▶); software used to prepare material for publication: *SHELXL97*.

## Supplementary Material

Crystal structure: contains datablock(s) I, global. DOI: 10.1107/S1600536812008707/bt5828sup1.cif

Structure factors: contains datablock(s) I. DOI: 10.1107/S1600536812008707/bt5828Isup2.hkl

Supplementary material file. DOI: 10.1107/S1600536812008707/bt5828Isup3.cdx

Supplementary material file. DOI: 10.1107/S1600536812008707/bt5828Isup4.cml

Additional supplementary materials:  crystallographic information; 3D view; checkCIF report

## Figures and Tables

**Table 1:** Hydrogen-bond geometry (Å, °)

*D*—H⋯*A*	*D*—H	H⋯*A*	*D*⋯*A*	*D*—H⋯*A*
N16—H16⋯Cl2	0.93	2.12	3.017 (3)	161
N18—H18⋯Cl1	0.93	2.16	3.078 (3)	171
O24—H24⋯Cl1	0.84	2.31	3.147 (3)	172
O22—H22⋯Cl2^i^	0.84	2.27	3.065 (3)	157
O23—H23⋯Cl2^ii^	0.84	2.36	3.169 (3)	163
C18—H18*A*⋯O22	0.99	2.23	2.924 (4)	126
C21—H21*A*⋯O23	0.99	2.39	3.266 (5)	147
C2—H2⋯F13*A*^iii^	0.95	2.45	3.381 (4)	165
C14—H14*A*⋯O23^ii^	0.99	2.51	3.482 (5)	169
C17—H17*A*⋯O24^iv^	0.99	2.24	3.215 (4)	166
C17—H17*B*⋯O22^i^	0.99	2.55	3.379 (5)	141
C16—H16*B*⋯Cl2^v^	0.99	2.67	3.619 (4)	161
C19—H19*B*⋯Cl2^ii^	0.99	2.75	3.529 (3)	136

Symmetry codes: (i) 
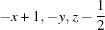
; (ii) 
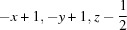
; (iii) 
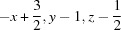
; (iv) 
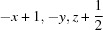
; (v) 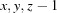.

**Fig. 1.**
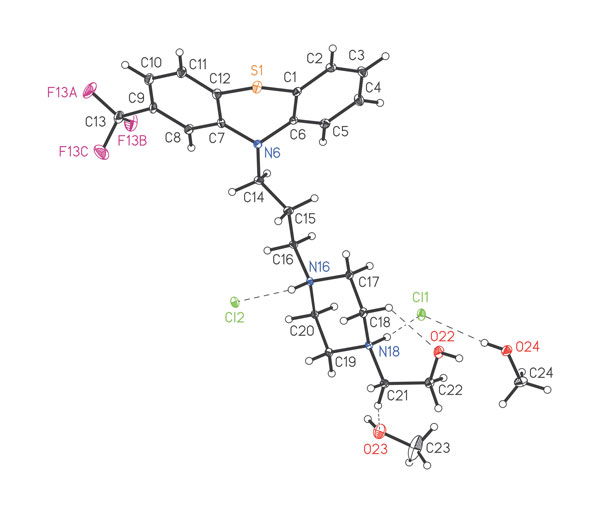
The structures and atom-numbering schemes for the FPh dication, chloride anions and solvent molecules joined by hydrogen bonds (dashed lines) in the asymmetric unit of I. Displacement ellipsoids are drawn at the 30% probability level.

**Fig. 2.**
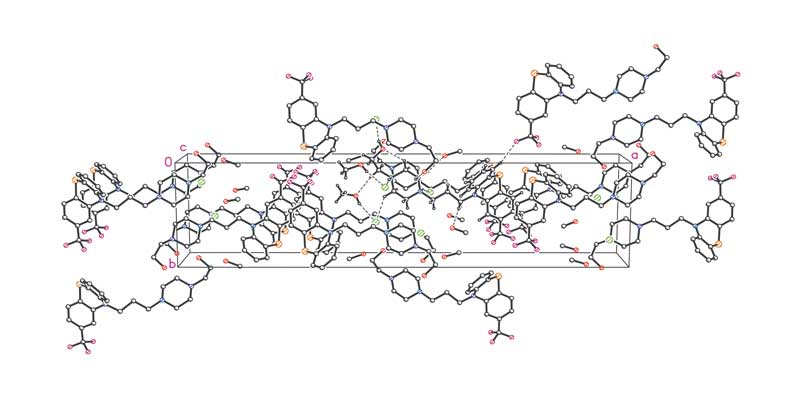
Packing diagram of the title compound viewed along the c axis.

## supplementary crystallographic information

### Comment

Fluphenazine (2-(4-(3-(2-(trifluoromethyl)-10*H*-phenothiazin-10-yl)propyl) piperazin-1-yl)ethanol)) (FPh) belongs to one of the oldest and the biggest family of antipsychotic drugs known as phenothiazines (Ohlow & Moosmann, 2011). Apart from its application in the treatment of many psychoses (mainly schizophrenia, mania and paranoid syndromes), it exhibits also a broad spectrum of biological effects, among them the anti-MDR (multidrug resistance) potency. (Gasiorowski *et al.*, 2001; Szabó *et al.*, 1999). Due to the anti-MDR activity of phenothiazines is strictly correlated with their structure (Tsakovska & Pajeva, 2006; Ford *et al.*, 1988), the aim of our work is to characterize the solid state structure of fluphenazine. In the crystal structure of I (Fig. 1), the dihedral angle between the planes of the two outer benzene rings of the phenothiazine system known as 'butterfly angle', correlates with values find for phenothiazines with high biological activity (Dahl *et al.*, 1986; McDowell, 1978; Yathirajan *et al.*, 2007). The piperazine ring adopts a chair conformation, as in the case before reported fluphenazine dipicrate (Dutkiewicz *et al.*, 2010), described by the Cremer & Pople (1975) puckering parameters *q*_2_ = 0.019 Å, *φ*_2_ = 13.9°, *q*_3_ = -0.593 Å, *Q* = 0.593 Å, *θ* = 178.2°. The crystal structure is stabilized by O—H···Cl, N—H···Cl, C—H···O, C—H···Cl and C—H···F hydrogen bonds and contacts (Table 1 and Fig. 2), that are very similar to those in trifluperazine dihydrochloride (McDowell, 1980).

### Experimental

The FPh^2+.^2Cl^-.^2CH_3_OH crystals were obtained by slow evaporation of methanol solution of dihydrochloride fluphenazine (Jelfa) at -15°C.

### Refinement

All H atoms were found in difference Fourier maps. In the final refinement cycles, all H atoms were positioned geometrically and treated as riding atoms, with C—H = 0.95–0.99 Å, N—H = 0.93 Å and O—H = 0.84 Å, and with *U*_iso_(H) = 1.2*U*_eq_(C, Nsp^3^) or 1.5*U*_eq_(O, C_methyl_).

### Crystal data

**Table:**

C_22_H_28_F_3_N_3_OS^2+^·2Cl^−^·2(CH_4_O)	*F*(000) = 1208
*M**_r_* = 574.53	*D*_x_ = 1.353 Mg m^−^^3^
Orthorhombic, *P**c**a*2_1_	Mo *K*α radiation, λ = 0.71073 Å
Hall symbol: P 2c -2ac	Cell parameters from 13454 reflections
*a* = 39.76 (2) Å	θ = 4.8–38.5°
*b* = 9.952 (8) Å	µ = 0.35 mm^−^^1^
*c* = 7.127 (5) Å	*T* = 85 K
*V* = 2820 (3) Å^3^	Needle, colourless
*Z* = 4	0.24 × 0.02 × 0.01 mm

### Data collection

**Table:**

Oxford Diffraction Xcalibur PX κ-geometry diffractometer with CCD Onyx camera	13922 independent reflections
Radiation source: fine-focus sealed tube	10615 reflections with *I* > 2σ(*I*)
Graphite/ monochromator	*R*_int_ = 0.052
ω and φ scans	θ_max_ = 38.6°, θ_min_ = 4.8°
Absorption correction: multi-scan (*CrysAlis RED*; Oxford Diffraction, 2007)	*h* = −69→64
*T*_min_ = 0.850, *T*_max_ = 1.000	*k* = −17→15
43952 measured reflections	*l* = −12→10

### Refinement

**Table:**

Refinement on *F*^2^	Secondary atom site location: difference Fourier map
Least-squares matrix: full	Hydrogen site location: inferred from neighbouring sites
*R*[*F*^2^ > 2σ(*F*^2^)] = 0.084	H-atom parameters constrained
*wR*(*F*^2^) = 0.197	*w* = 1/[σ^2^(*F*_o_^2^) + (0.062*P*)^2^ + 4.274*P*] where *P* = (*F*_o_^2^ + 2*F*_c_^2^)/3
*S* = 1.19	(Δ/σ)_max_ = 0.001
13922 reflections	Δρ_max_ = 1.25 e Å^−^^3^
330 parameters	Δρ_min_ = −0.85 e Å^−^^3^
1 restraint	Absolute structure: Flack (1983), 5579 Friedel pairs
Primary atom site location: structure-invariant direct methods	Flack parameter: 0.09 (7)

### Special details

**Table:**

Geometry. All e.s.d.'s (except the e.s.d. in the dihedral angle between two l.s. planes) are estimated using the full covariance matrix. The cell e.s.d.'s are taken into account individually in the estimation of e.s.d.'s in distances, angles and torsion angles; correlations between e.s.d.'s in cell parameters are only used when they are defined by crystal symmetry. An approximate (isotropic) treatment of cell e.s.d.'s is used for estimating e.s.d.'s involving l.s. planes.
Refinement. Refinement of *F*^2^ against ALL reflections. The weighted *R*-factor *wR* and goodness of fit *S* are based on *F*^2^, conventional *R*-factors *R* are based on *F*, with *F* set to zero for negative *F*^2^. The threshold expression of *F*^2^ > σ(*F*^2^) is used only for calculating *R*-factors(gt) *etc*. and is not relevant to the choice of reflections for refinement. *R*-factors based on *F*^2^ are statistically about twice as large as those based on *F*, and *R*- factors based on ALL data will be even larger.

### Fractional atomic coordinates and isotropic or equivalent isotropic displacement parameters (Å^2^)

**Table:**

	*x*	*y*	*z*	*U*_iso_*/*U*_eq_	
S1	0.716794 (19)	0.15955 (8)	0.47197 (13)	0.01988 (15)	
C1	0.69059 (7)	0.1626 (3)	0.2720 (4)	0.0141 (5)	
C2	0.69591 (8)	0.0733 (3)	0.1245 (5)	0.0186 (6)	
H2	0.7139	0.0109	0.1301	0.022*	
C3	0.67489 (8)	0.0756 (3)	−0.0306 (5)	0.0217 (6)	
H3	0.6781	0.0136	−0.1303	0.026*	
C4	0.64910 (8)	0.1692 (3)	−0.0392 (5)	0.0218 (5)	
H4	0.6347	0.1709	−0.1456	0.026*	
C5	0.64397 (7)	0.2604 (3)	0.1050 (5)	0.0178 (5)	
H5	0.6265	0.3251	0.0956	0.021*	
C6	0.66444 (7)	0.2571 (3)	0.2641 (4)	0.0131 (4)	
N6	0.66034 (6)	0.3468 (2)	0.4191 (4)	0.0154 (5)	
C7	0.68982 (7)	0.4120 (3)	0.4788 (4)	0.0146 (5)	
C8	0.69142 (8)	0.5497 (3)	0.5105 (5)	0.0176 (5)	
H8	0.6722	0.6045	0.4893	0.021*	
C9	0.72137 (8)	0.6071 (3)	0.5736 (5)	0.0204 (6)	
C10	0.75000 (9)	0.5322 (3)	0.5989 (5)	0.0219 (6)	
H10	0.7702	0.5731	0.6409	0.026*	
C11	0.74876 (8)	0.3957 (3)	0.5618 (5)	0.0208 (6)	
H11	0.7686	0.3432	0.5746	0.025*	
C12	0.71912 (7)	0.3344 (3)	0.5065 (4)	0.0174 (5)	
C13	0.72107 (9)	0.7549 (4)	0.6146 (6)	0.0288 (8)	
F13A	0.75142 (7)	0.8068 (2)	0.6355 (6)	0.0553 (10)	
F13B	0.70499 (8)	0.8260 (2)	0.4841 (5)	0.0470 (7)	
F13C	0.70460 (9)	0.7820 (3)	0.7761 (5)	0.0500 (8)	
C14	0.62843 (7)	0.4191 (3)	0.4335 (5)	0.0178 (5)	
H14A	0.6242	0.4684	0.3151	0.021*	
H14B	0.6299	0.4858	0.5363	0.021*	
C15	0.59930 (7)	0.3231 (3)	0.4715 (5)	0.0163 (5)	
H15A	0.5998	0.2482	0.3802	0.020*	
H15B	0.6013	0.2850	0.5993	0.020*	
C16	0.56640 (6)	0.4003 (2)	0.4535 (5)	0.0133 (4)	
H16A	0.5691	0.4910	0.5085	0.016*	
H16B	0.5607	0.4111	0.3192	0.016*	
N16	0.53816 (6)	0.3281 (2)	0.5520 (3)	0.0112 (4)	
H16	0.5436	0.3238	0.6788	0.013*	
C17	0.53309 (7)	0.1872 (3)	0.4842 (4)	0.0142 (5)	
H17A	0.5541	0.1354	0.5009	0.017*	
H17B	0.5276	0.1882	0.3488	0.017*	
C18	0.50516 (7)	0.1208 (3)	0.5911 (5)	0.0146 (5)	
H18A	0.5022	0.0278	0.5447	0.018*	
H18B	0.5113	0.1160	0.7256	0.018*	
N18	0.47276 (6)	0.1961 (2)	0.5704 (3)	0.0108 (4)	
H18	0.4676	0.2015	0.4433	0.013*	
C19	0.47808 (7)	0.3362 (3)	0.6430 (4)	0.0132 (4)	
H19A	0.4840	0.3327	0.7779	0.016*	
H19B	0.4570	0.3885	0.6296	0.016*	
C20	0.50609 (6)	0.4044 (2)	0.5348 (4)	0.0101 (4)	
H20A	0.4997	0.4111	0.4008	0.012*	
H20B	0.5093	0.4967	0.5835	0.012*	
C21	0.44365 (7)	0.1309 (3)	0.6692 (4)	0.0150 (5)	
H21A	0.4259	0.1990	0.6905	0.018*	
H21B	0.4512	0.0981	0.7933	0.018*	
C22	0.42902 (8)	0.0152 (3)	0.5600 (5)	0.0170 (5)	
H22A	0.4094	−0.0220	0.6277	0.020*	
H22B	0.4214	0.0468	0.4355	0.020*	
O22	0.45382 (7)	−0.0860 (2)	0.5376 (4)	0.0262 (5)	
H22	0.4445	−0.1616	0.5324	0.039*	
O23	0.39180 (8)	0.3725 (3)	0.5576 (5)	0.0333 (6)	
H23	0.4001	0.4451	0.5190	0.050*	
C23	0.36311 (18)	0.3410 (7)	0.4515 (13)	0.071 (2)	
H23A	0.3484	0.4200	0.4441	0.106*	
H23B	0.3510	0.2669	0.5115	0.106*	
H23C	0.3699	0.3142	0.3247	0.106*	
O24	0.40656 (6)	0.0214 (2)	0.0563 (4)	0.0233 (5)	
H24	0.4190	0.0875	0.0808	0.035*	
C24	0.37321 (9)	0.0675 (5)	0.0181 (6)	0.0335 (9)	
H24A	0.3639	0.1101	0.1306	0.050*	
H24B	0.3591	−0.0090	−0.0179	0.050*	
H24C	0.3738	0.1328	−0.0847	0.050*	
Cl1	0.459485 (19)	0.24652 (8)	0.15078 (11)	0.01844 (13)	
Cl2	0.562033 (17)	0.38032 (7)	0.94712 (10)	0.01575 (12)	

### Atomic displacement parameters (Å^2^)

**Table:**

	*U*^11^	*U*^22^	*U*^33^	*U*^12^	*U*^13^	*U*^23^
S1	0.0209 (3)	0.0132 (3)	0.0255 (4)	0.0016 (2)	−0.0071 (3)	0.0018 (3)
C1	0.0136 (11)	0.0084 (10)	0.0203 (13)	−0.0007 (9)	−0.0001 (9)	0.0013 (9)
C2	0.0164 (11)	0.0105 (11)	0.0289 (17)	0.0025 (9)	0.0035 (11)	−0.0022 (10)
C3	0.0259 (13)	0.0161 (12)	0.0230 (15)	0.0017 (10)	0.0032 (13)	−0.0057 (12)
C4	0.0257 (13)	0.0220 (13)	0.0175 (13)	0.0010 (11)	−0.0047 (12)	−0.0027 (13)
C5	0.0160 (12)	0.0170 (12)	0.0203 (14)	0.0028 (10)	−0.0017 (10)	−0.0001 (10)
C6	0.0135 (10)	0.0061 (9)	0.0198 (13)	−0.0007 (8)	0.0023 (9)	−0.0009 (9)
N6	0.0128 (9)	0.0112 (9)	0.0223 (13)	0.0003 (8)	0.0018 (8)	−0.0045 (8)
C7	0.0162 (10)	0.0123 (10)	0.0153 (12)	0.0000 (8)	−0.0009 (9)	−0.0019 (9)
C8	0.0164 (11)	0.0110 (11)	0.0253 (15)	−0.0001 (9)	0.0000 (10)	−0.0015 (10)
C9	0.0211 (13)	0.0143 (12)	0.0258 (15)	−0.0048 (10)	−0.0021 (12)	−0.0064 (11)
C10	0.0200 (13)	0.0205 (14)	0.0253 (16)	−0.0021 (11)	−0.0043 (12)	−0.0034 (12)
C11	0.0181 (12)	0.0198 (14)	0.0246 (15)	0.0008 (10)	−0.0057 (11)	−0.0019 (12)
C12	0.0163 (11)	0.0175 (12)	0.0184 (14)	0.0012 (10)	−0.0041 (10)	−0.0004 (10)
C13	0.0258 (15)	0.0192 (15)	0.041 (2)	−0.0062 (12)	0.0014 (14)	−0.0085 (15)
F13A	0.0261 (11)	0.0234 (11)	0.116 (3)	−0.0107 (10)	0.0014 (16)	−0.0251 (15)
F13B	0.0628 (17)	0.0125 (9)	0.066 (2)	−0.0013 (10)	−0.0150 (16)	−0.0016 (11)
F13C	0.066 (2)	0.0277 (13)	0.0561 (19)	−0.0046 (13)	0.0231 (16)	−0.0180 (13)
C14	0.0139 (10)	0.0171 (12)	0.0224 (14)	0.0014 (9)	0.0035 (11)	−0.0034 (11)
C15	0.0135 (10)	0.0122 (10)	0.0233 (14)	0.0015 (8)	0.0036 (10)	−0.0010 (11)
C16	0.0135 (10)	0.0110 (10)	0.0153 (11)	0.0004 (7)	0.0025 (10)	−0.0001 (10)
N16	0.0117 (9)	0.0114 (9)	0.0104 (9)	0.0011 (7)	−0.0015 (7)	−0.0008 (8)
C17	0.0143 (10)	0.0095 (10)	0.0187 (13)	0.0009 (8)	−0.0010 (9)	−0.0022 (9)
C18	0.0161 (11)	0.0062 (9)	0.0216 (13)	0.0014 (8)	−0.0022 (10)	−0.0014 (9)
N18	0.0133 (9)	0.0072 (8)	0.0118 (10)	0.0012 (7)	−0.0008 (8)	0.0006 (7)
C19	0.0155 (10)	0.0084 (9)	0.0156 (11)	−0.0011 (8)	0.0013 (9)	−0.0011 (9)
C20	0.0140 (10)	0.0037 (9)	0.0127 (11)	0.0007 (7)	−0.0002 (8)	0.0011 (7)
C21	0.0168 (11)	0.0126 (11)	0.0157 (12)	−0.0032 (9)	0.0031 (9)	0.0008 (9)
C22	0.0212 (12)	0.0118 (11)	0.0179 (13)	−0.0033 (9)	0.0008 (10)	0.0016 (10)
O22	0.0263 (11)	0.0120 (9)	0.0404 (15)	−0.0039 (8)	0.0014 (11)	−0.0060 (10)
O23	0.0375 (15)	0.0228 (13)	0.0394 (17)	−0.0038 (11)	−0.0043 (13)	0.0034 (12)
C23	0.080 (4)	0.055 (3)	0.077 (4)	−0.036 (3)	−0.038 (4)	0.013 (3)
O24	0.0189 (10)	0.0157 (10)	0.0353 (14)	−0.0021 (8)	0.0012 (10)	−0.0041 (10)
C24	0.0175 (14)	0.045 (2)	0.038 (2)	0.0028 (15)	0.0015 (14)	−0.0069 (17)
Cl1	0.0254 (3)	0.0182 (3)	0.0118 (2)	−0.0055 (3)	−0.0041 (3)	0.0017 (2)
Cl2	0.0212 (3)	0.0131 (2)	0.0129 (3)	0.0022 (2)	−0.0034 (2)	−0.0012 (2)

### Geometric parameters (Å, º)

**Table:**

S1—C12	1.760 (4)	C16—H16B	0.9900
S1—C1	1.766 (3)	N16—C20	1.489 (3)
C1—C2	1.393 (4)	N16—C17	1.497 (4)
C1—C6	1.403 (4)	N16—H16	0.9300
C2—C3	1.386 (5)	C17—C18	1.500 (4)
C2—H2	0.9500	C17—H17A	0.9900
C3—C4	1.387 (4)	C17—H17B	0.9900
C3—H3	0.9500	C18—N18	1.498 (4)
C4—C5	1.386 (5)	C18—H18A	0.9900
C4—H4	0.9500	C18—H18B	0.9900
C5—C6	1.396 (4)	N18—C19	1.502 (4)
C5—H5	0.9500	N18—C21	1.502 (4)
C6—N6	1.430 (4)	N18—H18	0.9300
N6—C7	1.406 (4)	C19—C20	1.516 (4)
N6—C14	1.462 (4)	C19—H19A	0.9900
C7—C8	1.390 (4)	C19—H19B	0.9900
C7—C12	1.412 (4)	C20—H20A	0.9900
C8—C9	1.395 (4)	C20—H20B	0.9900
C8—H8	0.9500	C21—C22	1.507 (4)
C9—C10	1.373 (5)	C21—H21A	0.9900
C9—C13	1.499 (5)	C21—H21B	0.9900
C10—C11	1.385 (5)	C22—O22	1.418 (4)
C10—H10	0.9500	C22—H22A	0.9900
C11—C12	1.385 (4)	C22—H22B	0.9900
C11—H11	0.9500	O22—H22	0.8400
C13—F13A	1.321 (4)	O23—C23	1.404 (7)
C13—F13B	1.332 (5)	O23—H23	0.8400
C13—F13C	1.352 (5)	C23—H23A	0.9800
C14—C15	1.526 (4)	C23—H23B	0.9800
C14—H14A	0.9900	C23—H23C	0.9800
C14—H14B	0.9900	O24—C24	1.429 (4)
C15—C16	1.522 (4)	O24—H24	0.8400
C15—H15A	0.9900	C24—H24A	0.9800
C15—H15B	0.9900	C24—H24B	0.9800
C16—N16	1.507 (4)	C24—H24C	0.9800
C16—H16A	0.9900		
			
C12—S1—C1	97.29 (14)	C20—N16—C17	109.6 (2)
C2—C1—C6	120.6 (3)	C20—N16—C16	110.9 (2)
C2—C1—S1	120.6 (2)	C17—N16—C16	113.4 (2)
C6—C1—S1	118.8 (2)	C20—N16—H16	107.6
C3—C2—C1	120.0 (3)	C17—N16—H16	107.6
C3—C2—H2	120.0	C16—N16—H16	107.6
C1—C2—H2	120.0	N16—C17—C18	110.4 (2)
C2—C3—C4	119.5 (3)	N16—C17—H17A	109.6
C2—C3—H3	120.3	C18—C17—H17A	109.6
C4—C3—H3	120.3	N16—C17—H17B	109.6
C5—C4—C3	121.1 (3)	C18—C17—H17B	109.6
C5—C4—H4	119.5	H17A—C17—H17B	108.1
C3—C4—H4	119.5	N18—C18—C17	111.5 (2)
C4—C5—C6	120.1 (3)	N18—C18—H18A	109.3
C4—C5—H5	120.0	C17—C18—H18A	109.3
C6—C5—H5	120.0	N18—C18—H18B	109.3
C5—C6—C1	118.7 (3)	C17—C18—H18B	109.3
C5—C6—N6	123.1 (3)	H18A—C18—H18B	108.0
C1—C6—N6	118.2 (3)	C18—N18—C19	108.0 (2)
C7—N6—C6	115.3 (2)	C18—N18—C21	113.6 (2)
C7—N6—C14	118.4 (2)	C19—N18—C21	110.3 (2)
C6—N6—C14	117.4 (2)	C18—N18—H18	108.2
C8—C7—N6	122.8 (3)	C19—N18—H18	108.2
C8—C7—C12	118.6 (3)	C21—N18—H18	108.2
N6—C7—C12	118.6 (3)	N18—C19—C20	110.1 (2)
C7—C8—C9	119.7 (3)	N18—C19—H19A	109.6
C7—C8—H8	120.2	C20—C19—H19A	109.6
C9—C8—H8	120.2	N18—C19—H19B	109.6
C10—C9—C8	121.9 (3)	C20—C19—H19B	109.6
C10—C9—C13	120.9 (3)	H19A—C19—H19B	108.1
C8—C9—C13	117.2 (3)	N16—C20—C19	111.0 (2)
C9—C10—C11	118.6 (3)	N16—C20—H20A	109.4
C9—C10—H10	120.7	C19—C20—H20A	109.4
C11—C10—H10	120.7	N16—C20—H20B	109.4
C12—C11—C10	121.1 (3)	C19—C20—H20B	109.4
C12—C11—H11	119.5	H20A—C20—H20B	108.0
C10—C11—H11	119.5	N18—C21—C22	112.7 (2)
C11—C12—C7	120.1 (3)	N18—C21—H21A	109.1
C11—C12—S1	121.3 (2)	C22—C21—H21A	109.1
C7—C12—S1	118.6 (2)	N18—C21—H21B	109.1
F13A—C13—F13B	108.0 (4)	C22—C21—H21B	109.1
F13A—C13—F13C	105.6 (3)	H21A—C21—H21B	107.8
F13B—C13—F13C	104.8 (3)	O22—C22—C21	109.4 (3)
F13A—C13—C9	113.5 (3)	O22—C22—H22A	109.8
F13B—C13—C9	112.9 (3)	C21—C22—H22A	109.8
F13C—C13—C9	111.4 (3)	O22—C22—H22B	109.8
N6—C14—C15	111.3 (2)	C21—C22—H22B	109.8
N6—C14—H14A	109.4	H22A—C22—H22B	108.2
C15—C14—H14A	109.4	C22—O22—H22	109.5
N6—C14—H14B	109.4	C23—O23—H23	109.5
C15—C14—H14B	109.4	O23—C23—H23A	109.5
H14A—C14—H14B	108.0	O23—C23—H23B	109.5
C16—C15—C14	108.8 (2)	H23A—C23—H23B	109.5
C16—C15—H15A	109.9	O23—C23—H23C	109.5
C14—C15—H15A	109.9	H23A—C23—H23C	109.5
C16—C15—H15B	109.9	H23B—C23—H23C	109.5
C14—C15—H15B	109.9	C24—O24—H24	109.5
H15A—C15—H15B	108.3	O24—C24—H24A	109.5
N16—C16—C15	111.1 (2)	O24—C24—H24B	109.5
N16—C16—H16A	109.4	H24A—C24—H24B	109.5
C15—C16—H16A	109.4	O24—C24—H24C	109.5
N16—C16—H16B	109.4	H24A—C24—H24C	109.5
C15—C16—H16B	109.4	H24B—C24—H24C	109.5
H16A—C16—H16B	108.0		
			
C12—S1—C1—C2	141.0 (3)	N6—C7—C12—C11	178.8 (3)
C12—S1—C1—C6	−38.6 (3)	C8—C7—C12—S1	178.4 (2)
C6—C1—C2—C3	−1.2 (4)	N6—C7—C12—S1	−2.0 (4)
S1—C1—C2—C3	179.2 (2)	C1—S1—C12—C11	−141.1 (3)
C1—C2—C3—C4	1.4 (5)	C1—S1—C12—C7	39.8 (3)
C2—C3—C4—C5	−0.1 (5)	C10—C9—C13—F13A	13.7 (6)
C3—C4—C5—C6	−1.4 (5)	C8—C9—C13—F13A	−166.8 (4)
C4—C5—C6—C1	1.6 (4)	C10—C9—C13—F13B	137.1 (4)
C4—C5—C6—N6	−179.2 (3)	C8—C9—C13—F13B	−43.4 (5)
C2—C1—C6—C5	−0.3 (4)	C10—C9—C13—F13C	−105.3 (4)
S1—C1—C6—C5	179.3 (2)	C8—C9—C13—F13C	74.2 (5)
C2—C1—C6—N6	−179.5 (3)	C7—N6—C14—C15	−148.0 (3)
S1—C1—C6—N6	0.1 (4)	C6—N6—C14—C15	66.1 (4)
C5—C6—N6—C7	−130.1 (3)	N6—C14—C15—C16	−170.9 (3)
C1—C6—N6—C7	49.0 (4)	C14—C15—C16—N16	−160.3 (3)
C5—C6—N6—C14	16.7 (4)	C15—C16—N16—C20	179.7 (2)
C1—C6—N6—C14	−164.1 (3)	C15—C16—N16—C17	−56.5 (3)
C6—N6—C7—C8	131.6 (3)	C20—N16—C17—C18	−56.8 (3)
C14—N6—C7—C8	−14.9 (5)	C16—N16—C17—C18	178.8 (2)
C6—N6—C7—C12	−47.9 (4)	N16—C17—C18—N18	59.1 (3)
C14—N6—C7—C12	165.6 (3)	C17—C18—N18—C19	−59.3 (3)
N6—C7—C8—C9	178.6 (3)	C17—C18—N18—C21	177.9 (2)
C12—C7—C8—C9	−1.9 (5)	C18—N18—C19—C20	58.7 (3)
C7—C8—C9—C10	2.6 (5)	C21—N18—C19—C20	−176.6 (2)
C7—C8—C9—C13	−177.0 (3)	C17—N16—C20—C19	57.3 (3)
C8—C9—C10—C11	−0.6 (6)	C16—N16—C20—C19	−176.8 (2)
C13—C9—C10—C11	179.0 (3)	N18—C19—C20—N16	−59.3 (3)
C9—C10—C11—C12	−2.1 (6)	C18—N18—C21—C22	−79.5 (3)
C10—C11—C12—C7	2.8 (5)	C19—N18—C21—C22	159.0 (2)
C10—C11—C12—S1	−176.4 (3)	N18—C21—C22—O22	61.8 (3)
C8—C7—C12—C11	−0.7 (5)		

### Hydrogen-bond geometry (Å, º)

**Table:**

*D*—H···*A*	*D*—H	H···*A*	*D*···*A*	*D*—H···*A*
N16—H16···Cl2	0.93	2.12	3.017 (3)	161
N18—H18···Cl1	0.93	2.16	3.078 (3)	171
O24—H24···Cl1	0.84	2.31	3.147 (3)	172
O22—H22···Cl2^i^	0.84	2.27	3.065 (3)	157
O23—H23···Cl2^ii^	0.84	2.36	3.169 (3)	163
C18—H18*A*···O22	0.99	2.23	2.924 (4)	126
C21—H21*A*···O23	0.99	2.39	3.266 (5)	147
C2—H2···F13*A*^iii^	0.95	2.45	3.381 (4)	165
C14—H14*A*···O23^ii^	0.99	2.51	3.482 (5)	169
C17—H17*A*···O24^iv^	0.99	2.24	3.215 (4)	166
C17—H17*B*···O22^i^	0.99	2.55	3.379 (5)	141
C16—H16*B*···Cl2^v^	0.99	2.67	3.619 (4)	161
C19—H19*B*···Cl2^ii^	0.99	2.75	3.529 (3)	136

Symmetry codes: (i) −*x*+1, −*y*, *z*−1/2; (ii) −*x*+1, −*y*+1, *z*−1/2; (iii) −*x*+3/2, *y*−1, *z*−1/2; (iv) −*x*+1, −*y*, *z*+1/2; (v) *x*, *y*, *z*−1.
